# Filling Temporary Teeth

**Published:** 1876-08

**Authors:** J. T. Abbott


### FILLING TEMPORARY TEETH.

BY DR. J. T. ABBOTT.

*Read before the Iowa State Dental Society.*

The great trouble in the West has been and is to get the temporary teeth filled. Our patients are being educated in the direction for the better every year. The rule is the child is not brought to us until the tooth is aching. The pulp is exposed or perhaps dead and abscessed at the roots. Then we are expected to relieve the pain at once and save the tooth. Every dentist is cognizant of the fact that the preservation of the temporary teeth has much to do with the future well-being of the permanent ones, and when this fact can be so firmly impressed upon the mind of the parents as to induce them to have the children's teeth examined and operated upon as the case may require, then we shall have less abuses to contend with and less aching teeth; mothers will not be worn out by sleepless nights caused by children crying with the tooth ache, and the permanent teeth are more likely to appear in a more healthy or normal condition. The simple aching or loss of the temporary teeth is not the worst feature of the case. Temporary aching teeth, diseased teeth, abscessed teeth often induce a train of constitutional abnormal results, for "nature once obstructed often breeds frightful results."

Temporary teeth usually yield more kindly to judicious treatment than permanent ones. I have found that exposed pulps may be cared for and capped and the teeth filled with success, when the same treatment with permanent teeth would prove a failure. The vital forces are more active, the absorbents from the very nature of the case are judiciously attending to their office work.

The materials for filling can not positively be designated.

My practice has been to use amalgam washed, but so much has been said about the shrinking of washed amalgam (and apparently proved by Dr. Bogue of New York and others) that I am almost afraid to wash my amalgam any more, but am induced to occasionally, carelessly, to put in an unwashed filling, oxide and all. If the unwashed theory proves good practice, we certainly risk nothing in using it thus in temporary teeth for the retention of the filling is but temporary. Material that will do in one case may not in another. "Hill's stopping," "gutta percha," "oxychloride," and "Guilloi's cement," and other materials may be used, the good judgment of the operator guiding him. I think very much of Guilloi's cement for frail, temporary teeth. The principal objection to it is that it takes time to harden, and we all know how diffiit is to control our little patients. I do not deem it necessary to remove all the decayed portion of the tooth at the part of the cavity nearest the pulp as perhaps it is in the permanent teeth, so that the margins are well cleaned and all sharp edges removed. Then any of the fillings usually used if the walls be made impervious to fluids and the plugs will not wear or wash away will accomplish the end sought. I

think it will usually do (when a portion of caries is left in the cavity for protection, or for fear of coming in contact with the pulp) to apply carbolic acid for the purpose of destroying parasitic action. Leaving the acid in contact five minutes or more, the acidulated condition of the system will often counteract the best directed efforts of the dentist; in such cases, consult with the family physician. When there is but superficial decay on the approximal surfaces of the teeth, filing and polishing the same will often prevent further decay; but such teeth are frequently of frail texture and the filed surface leaves broad soft dentine to be acted upon by the acid solutions of the mouth. I must confess that filling in my practice of temporary teeth has been successful only in a comparatively few cases, hence if approximal cavities are susceptible of filling with but slight removal of good dentine I prefer the latter course. Another reason for this course is the carelessness of the little ones in keeping appointments and giving us opportunities for frequent examinations, and also the neglect of the little ones to keep the filled surfaces cleaned.

